# Alternatives for sustained disaster risk reduction: A re-assessment

**DOI:** 10.4102/jamba.v15i1.1487

**Published:** 2023-07-26

**Authors:** Loic Le De, Louise L. Baumann, Annabelle Moatty, Virginie Le Masson, Faten Kikano, Mahmood Fayazi, Manuela Fernandez, Isabella Tomassi, Jake Rom D. Cadag

**Affiliations:** 1 School of Public Health and Interdisciplinary Studies, Auckland University of Technology, Auckland, New Zealand; 2 School of Environment, Faculty of Science, Waipapa Taumata Rau University of Auckland, Auckland, New Zealand; 3 Department of Sismology, Institut de Physique du Globe de Paris – IPGP, Université Paris Cité, Centre National de la Recherche Scientifique – CNRS, Paris, France; 4 Institute for Risk and Disaster Reduction, University College London, London, United Kingdom; 5 Centre d’étude en responsabilité sociale et écocitoyenneté (CÉRSÉ), Collège de Rosemont, Montreal, Canada; 6 Architecture Sans Frontières International, Paris, France; 7 Centre RISC, Institution: Collège Notre-Dame-de-Foy, Québec, Canada; and, École d’Architecture, Aménagement, Université de Montréal, Montréal, Canada; 8 Conicet National Scientific and Technical Research Council, San Carlos de Bariloche, Argentina; 9 INTA – National Institute of Agricultural Technology, San Carlos de Bariloche, Argentina; 10 Géographie, UFR Temps et Territoires, EVS, Université Lumières Lyon 2, Lyon, France; 11 Department of Geography, College of Social Sciences and Philosophy, University of the Philippines Diliman, Quezon City, Philippines

**Keywords:** disaster risk reduction, vulnerability, paradigm, Francophone, disaster studies

## Abstract

Alternatives for sustained disaster risk reduction’ was published in 2010 by Francophone and Anglophone researchers as a critique on the way disasters were studied and disaster risk reduction handled in the Francophone sphere. The authors criticized the dominant Francophone approach for being heavily hazard-centred and called for more emphasis on vulnerability to understand disasters and foster disaster risk reduction – a shift that had already taken place in the Anglophone disaster literature. Twelve years later, this paper draws upon a bibliographic analysis to examine if the arguments developed in the 2010 publication have stem attention in the Francophone disaster literature.

**Contribution:**

The article finds that the shift towards the vulnerability paradigm has, to some extent, happened but took much longer in the French context than in the Spanish language and the Asian disaster literature. The article emphasises the need for a re-assessment of our practices and study of disasters, including reflections on what disasters are studied, how, by whom, and for whom. Eventually, alternatives for sustained disaster risk reduction now and in the future might include drawing upon more diverse ontologies and epistemologies that are pertinent locally, considering local people as co-researchers though participatory methods, and empowering local Francophone researchers to play a greater role in researching disasters and leading disaster risk reduction in their own localities.

## Introduction

In 2010, several Francophone researchers with their Anglophone colleagues published an article entitled ‘Alternatives pour une réduction durable des risques de catastrophe’ [Alternatives for sustained disaster risk reduction] (Gaillard et al. 2010). The full version of the article was written in French accompanied by shorter English and Spanish texts. The article aimed at reaching a Francophone audience working in the disaster field and was published in Human Geography, which is now part of SAGE journals. It stemmed from the frustration that the authors experienced at the time because of the use of a paradigm that privileged natural hazards as both causes of disasters and as the focus of efforts to reduce disaster risk. The authors felt that Francophone scientists, institutions, governments and media focused on the extreme dimension of hazards to both explain the causes of disasters and develop actions for disaster risk reduction (DRR). Disasters were portrayed as rare and large events exceeding people’s capacities to deal with them. Disasters were thus seen as external ‘events’ disconnected from the daily socio-economic fabric of societies and seen to require extraordinary solutions informed by scientific experts.

This dominant approach in the Francophone world felt retrograde considering the evolutions of the disaster risk domain taking place elsewhere, particularly in the Anglophone, Spanish language and Asian literatures (Alexander 2002; Bankoff 2001; Delica-Willison & Willison 2004; ed. Hewitt 1983; Lavell 1992). Since the 1980s, several scientists, mainly anthropologists, sociologists and geographers, had documented how maldevelopment shapes people’s vulnerability and creates the conditions for a hazard to turn into a disaster – this has been termed the vulnerability paradigm. Eventually, the work from an informal network of social scientists studying disasters in Latin America (La Red) showed that the accumulated impacts of small-scale hazards often exceed that of larger-scale disasters that make the media headlines and steer the attention of scientists and organisations working in DRR (Garcia Acosta 1996; Maskrey 1993). ‘Alternatives for sustained disaster risk reduction’ expressed the need for a shift in the way disasters were researched and DRR fostered in the Francophone world where such ideas did not seem to have generated much traction. The article called for moving away from the dominant hazard-focused paradigm, towards an approach that reframes disasters and disaster risk accumulation in the context of people’s daily struggles, including a better understanding of vulnerabilities, marginalisation processes and failed development policies.

Twelve years later, it is questionable whether there has been any uptake of the ideas developed in the 2010’s publication in the Francophone literature. Google Scholar indeed suggests that the article has attracted little attention with a total of 57 citations, from which only 15 were cited by Francophone researchers. The objectives of this article are therefore:

- to examine whether ‘Alternatives for sustained disaster risk reduction’ and the arguments it developed have received attention in the Francophone disaster literature, including how and why,
- to discuss whether the arguments developed in the 2010’s publication are still valid nowadays and
- to explore what alternatives for sustained DRR are needed moving forward.

## ‘Alternatives for sustained disaster risk reduction’: Key arguments developed in the 2010’s publication

‘Alternatives for sustained disaster risk reduction’ developed four main arguments:

- the Francophone study of disaster places a huge emphasis on rare and extreme hazards to explain disasters – this has been termed the hazard paradigm;
- this paradigm results in actions that focus heavily on such hazards and are often highly technical, technocratic and top-down;
- disasters occur mainly because of people’s vulnerability and there is a need for Francophone researchers to shift more towards better appraising and addressing vulnerability processes;
- disasters are local issues and Francophone researchers, policy makers and practitioners should work towards understanding and building on local capacities for sustained DRR.

These arguments indeed reflect the critiques, theories and recommendations developed by certain social scientists from the late 1970s to the mid-2000s period (Bankoff 2001; ed. Hewitt 1983; Lewis 1999; Wisner 1993).

‘Alternatives’ start with a criticism of the hazard paradigm that has dominated DRR scientific research and policies for emphasising the importance of natural hazards. With this paradigm, disasters are understood first and foremost as the result of extreme and rare natural events surpassing people’s capability to overcome them (White 1974). This leads to disasters being studied and managed as extraordinary ‘events’ outside of normality. The regions, countries and populations affected are generally considered unable to cope with such natural forces and often said to be underdeveloped and underprepared (Burton, Kates & White 1978). This has produced a divide between the so-called ‘developing’ nations depicted as non-prepared, unsafe or even dangerous and the ‘developed’ countries portrayed as safer and better prepared (Bankoff 2001).

With the hazard paradigm, physical scientists such as geologists, volcanologists and engineers focus mainly on predicting, monitoring and modelling the probabilistic occurrence of natural hazards and their associated effects. At the same time, social scientists have focused on people’s perception of risk linked with hazards and how they ‘adjust’ in the face of these phenomena. People who have a low perception of risk are said to not adjust sufficiently to potential risks, while those with high-risk perception allegedly prepare well to face natural hazards (Burton et al. 1978). The hazard paradigm leads to highly technical measures usually involving engineering enhancements, development of new construction norms and building codes, stronger climate forecasting and technological improvements for early warning systems. In parallel, the study of risk-perception-and-adjustment has led social scientists to focus on the development of insurance schemes and communication strategies to raise awareness about the risks associated with natural hazards (Cutter et al. 2015; Kates 1971). Experts such as volcanologists, engineers, geologists, modellers or behavioural scientists are thus essential to DRR; they are necessary but, as we shall see, not sufficient. Another problem is that at the global level, such an approach has implied that the global North should transfer its knowledge, technology and expertise to the global South, often in a top-down fashion (Bankoff 2001; Hewitt 1995).

The hazard paradigm, encompassing the study of people’s perception and adjustment to natural hazards, has been extensively criticised for being too technocratic, often failing to address the root causes of disasters and leading to inadequate outcomes, including increasing disaster risk. In spite of the enormous efforts from the advocates of the hazard paradigm, the Center for Epidemiological Research on Disasters (CRED) database – a database used by researchers, policy makers and organisations involved in DRR to guide their actions – shows an increase in disasters since the second half of the 20th century.

‘Alternatives’ is one of the many analyses that have questioned the relevance of the hazard paradigm for DRR. The authors warned that the criteria utilised by the CRED (fatalities, economic impacts, etc.) place an emphasis on large-scale disasters and portray the ‘developing’ countries from the global South as particularly at risk and in need of external intervention (Bankoff 2001). The authors argued that large-scale disasters reported in the CRED database needed to be handled with care as they do not provide disaggregated assessments by gender, age or socio-economic origin, thus tend to homogenise the affected population. Disasters are primarily social events where impacts are never uniform. Some people are more affected compared to others despite being exposed to the same hazard. A key to understanding this unequal impact of hazards and disasters is the recognition of people’s vulnerabilities and the social processes that lead to them.

The concept of vulnerability has been actively explored since the 1970s. For example, in an article entitled ‘Taking the naturalness out of natural disasters’, O’Keefe, Westgate and Wisner (1976) highlighted the unequal impacts of disasters on those most vulnerable within society. Gaillard et al. (2010:68) state: ‘still today the poor, many women, children, elderly, those without shelter, handicapped, refugees, prisoners or members of ethnic minorities are forgotten in the analysis of disaster’. Vulnerability refers to the characteristics of a society that make a hazard likely to become a disaster (Wisner 2004). The vulnerability paradigm emphasises that disasters are socio-economic, historical and political in their origin. They are linked to the unequal and unfair access to resources among society members (ed. Hewitt 1983). People can be vulnerable in different ways, economically, socially, geographically, politically and often a combination of these (Wisner 1993). Vulnerability evolves through time and depends on both local and global drivers (Blaikie et al. 1994). Disasters are understood as amplifiers of people’s everyday hardships, including food insecurity, precarious or weak shelter, poor health conditions and poverty (Wisner 2016). It is thus paramount for researchers, policy makers and practitioners to both understand and tackle vulnerability to achieve sustained DRR.

The last argument ‘Alternatives’ advances concerns the need to recognise and support people’s capacities in the face of hazards and disasters. Capacities are generally said to be endogenous to local people and reflect their skills, knowledge and resources. Capacities and the related coping strategies are not extraordinary actions people display in the case of rare and extreme events but are generally rooted in their daily lives (Gaillard et al. 2010). It is therefore critical to reframe disasters and DRR in people’s everyday life context. Appraising capacities is, nonetheless, very difficult for outside scholars and practitioners as it requires a strong understanding of the local dynamics, including power relationships, customs and socio-cultural elements shaping such capacities. Participatory approaches and tools are thus critical as they build on people’s knowledge, skills and resources but also place them first in the analysis of and solutions to problems that affect their lives (Chambers 1994; Delica-Willison & Willison 2004). At the same time, participation of local people in DRR cannot happen in isolation from external aid agencies but requires resources and support at the national and/or international level. In other words, sustained DRR necessitates a combination of both the bottom-up and the top-down.

## Methodological approach: A bibliometric analysis of the Francophone disaster literature

Drawing a clear and comprehensive picture of the Francophone disaster literature is complex. The ‘Francophonie’ refers to all countries having in common the total or partial use of the French language. In addition to France and some of its European neighbours (e.g. Belgium, Switzerland, Luxembourg), French is spoken in Canada and in many former French or Belgian colonies in Western Africa such as Benin, Ivory Coast, Guinea, Mali, Niger and Senegal [not an exhaustive list]. To a lesser extent, French is also spoken in Southeast Asia (i.e. Vietnam), the Middle East (i.e. Lebanon) and the Pacific Island Region (i.e. Vanuatu). In order to get a better sense of the uptakes of the vulnerability paradigm in the French-speaking disaster field, we conducted a bibliometric analysis on three different academic platforms (Web of Science, Theses.fr and ProQuest), focusing on peer-reviewed publications and PhD theses published in French over the past 70 years. We mostly centred our analysis on the French and Canadian contexts. This is because our analysis shows that as of today, the North-South academic balance of power results in French-speaking African scholars producing a minority of French-speaking disaster literature globally. Only 1.8% (*n* = 50) of the publications are led by African researchers, most of them co-authoring with Western French speakers.

The Web of Science (WoS) database search involved using ‘TOPIC’, which includes title, abstract, author keywords and Keywords Plus. In addition, we applied a language filter to each of our searches in order to select only publications in French (LA=(French)). More precisely, the search of the WoS to determine a set of relevant texts was the following: (TS=(= (disaster* OR risk* OR tsunami* OR hurricane* OR earthquake* OR eruption* OR flood* OR cyclone*)) AND LA=(French). It allowed the extraction of 35 106 results from the Core Collection database before the sorting step by Research Areas. Once the sorting was completed, 2667 results remained.

For the Theses.fr scoping research, we carried out an advanced search on the French website theses.fr (listing 519 265 theses in February 2022) from 01 January 1985 to 31 December 2021. Only defended theses were included. We used ‘TITLE’ as the primary search field for the following words: ‘disaster*’, ‘tsunami*’, ‘hurricane*’’earthquake*’, ‘eruption*’, ‘flood*’ or ‘cyclone*’. We chose to exclude from our results theses published in medical sciences that we considered to be beyond the scope of this study. According to our criteria, 453 theses relating to disasters were defended in France between 1985 and 2021.

Lastly, an advanced search on the website ProQuest was carried out with documents published from 01 January 1990 to 28 February 2022. We used ‘TITLE’ as the primary search field for the following words: ‘disaster*’, ‘risk’*, ‘tsunami*’, ‘hurricane*’, ‘earthquake*’, ‘eruption*’, ‘flood*’ or ‘cyclone*’. In addition, we applied a document type, a language and a country filter in order to only select dissertations and theses published in French and in Canada this time period. It allowed for the extraction of 670 Canadian dissertations or theses published in French between 1990 and 2022.

## On the impact in Francophone disaster studies: The late emergence of the vulnerability paradigm

**FIGURE 1:**
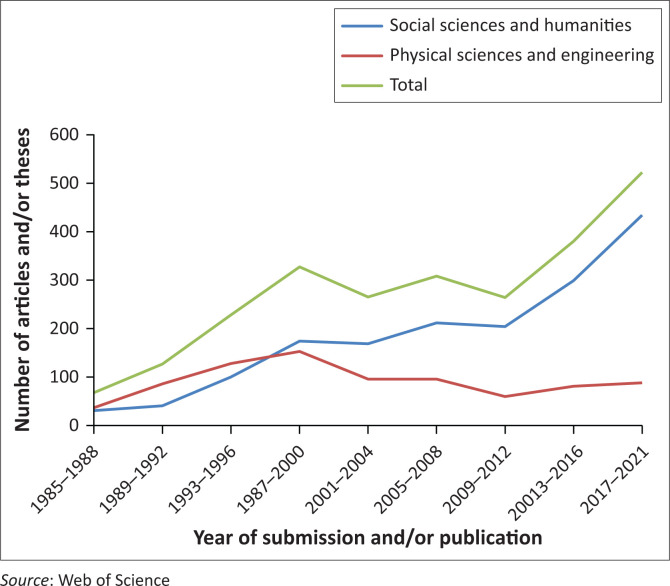
Yearly publications in French focusing on disasters from 1985^1^ to 2021.

**FIGURE 2:**
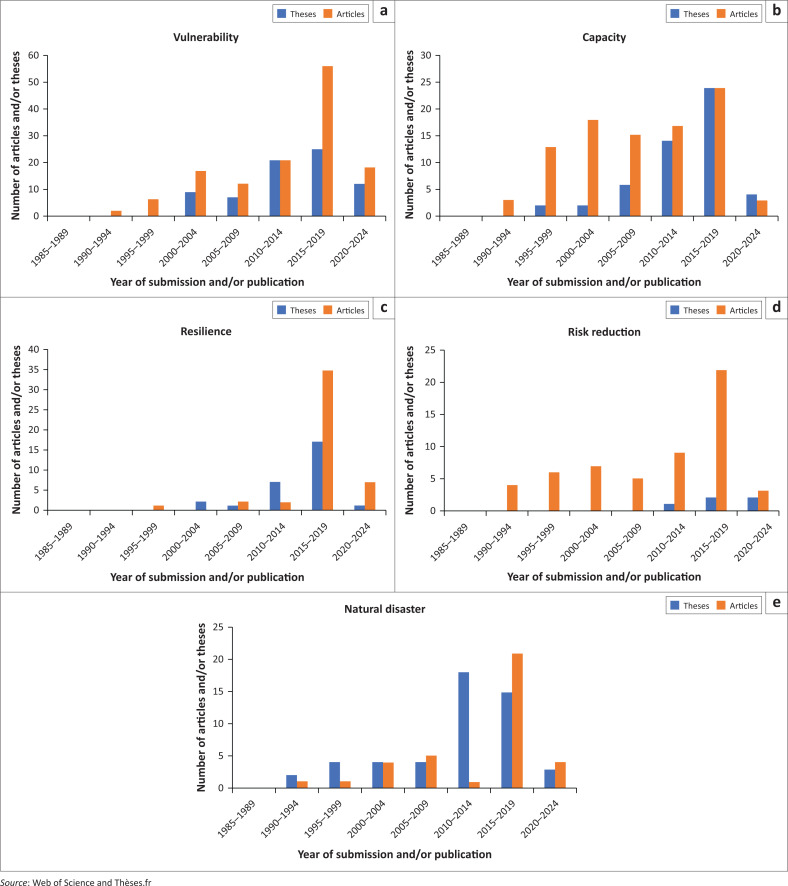
Evolution of the use of key concepts in French doctoral theses and articles from disaster studies (1985–2021).

The formal emergence of French-speaking^1^ disaster studies can roughly be dated back to the early 1990s (Figure 1). Since then, the number of disaster publications and PhD theses in French has increased significantly, with two research peaks in the late 1990s, and for the 2015–2019 period. At the same time, there is evidence that some of the core ideas of the vulnerability paradigm have progressed in the Francophone disaster literature. Both emerging and more established French-speaking researchers increasingly emphasise that disasters result from the unfair distribution of resources and power in society. Concepts like ‘vulnerability’, ‘capacity’ and ‘resilience’, strongly linked to the vulnerability paradigm, have gained in popularity since the early 2000s, with an important rise between 2010 and 2021 (Figure 2). Most of the theses (71%) and articles (68%) mentioning ‘vulnerability’, ‘capacity’ or ‘resilience’ in their abstract were from social sciences, which suggests that the increasing use of these concepts and progressive change of paradigm might be linked to the increasing number of Francophone social scientists working in disaster studies from the early 2000s (e.g. Leone & Vinet 2006; November, Penelas & Viot 2011; Pigeon 2012; Reghezza 2006; Revet 2011, 2013).

Nonetheless, while the emergence of the vulnerability paradigm in the English-speaking disaster context occurred in the late 1970s, this paradigmatic change only started to gain traction from around 2010 in the French context. The radical and political criticisms formulated and institutionalised in the 1970s in the Anglo-Saxon world penetrated the Latin American and South-African contexts as early as the mid-1990s (see Cabane & Revet 2015). This paradigm shift took much longer in the French context. Language barrier might have played a role. As of today, very few pioneer radical disaster texts and books have been translated into French. *At Risk: Natural Hazards, People’s Vulnerability and Disasters* (Blaikie et al. 1994), for instance, has still not been translated in French while it was translated in Spanish in 1996 and in Japanese in 2010. Another explanation could be that with the development of technology, the new generation of French-speaking scholars had, from the mid-2000s, greater access to a broader range of academic resources and were therefore more easily able to engage with the Anglophone disaster literature.

The increasing use of ‘vulnerability’, ‘resilience’ and ‘capacity’ is a good indicator of an increasing engagement of French-speaking doctoral candidates and disaster scholars with the core ideas of the vulnerability paradigm. However, these figures need to be analysed with care. The growing use of these concepts does not necessarily mean that their utilisation is in line with the historical radical claims of the school of thoughts from which they emerged. Furthermore, this trend needs to consider other tendencies. The continuing use of the term ‘natural disaster’, for instance, reflects persistence of the logic of the hazard paradigm. While the advocates of the vulnerability paradigm strongly reject the expression, encouraging scholars to document the unnatural, anthropogenic, socio-political dimension of disasters (Bankoff 2010; Chmutina & Von Meding 2019; O’Keefe et al. 1976), a very large number of Francophone disaster scholars keep using this expression. It was found that almost half (46%) of the 76 doctoral theses containing the term ‘disaster’ within their title defended since 2011 did use the expression ‘natural disaster’ in their abstract. In comparison, the term ‘risk reduction’ – a strong marker of the vulnerability paradigm that embodies the logic that simply ‘managing’ disastrous events is not sufficient, and that reducing risk through systemic efforts is indeed possible – only represents 1% (*n* = 5) of the 453 theses and 2% of the 2667 articles referring to it in their abstract.

Overall, the publications mentioning ‘vulnerability’, ‘resilience’ or ‘capacity’ only represent 20.4% of the publications found in our review. This illustrates well the limits of the increasing engagement of French-speaking disaster scholars with the ideas of the vulnerability paradigm. These limits are further revealed by the lack of research published in French analysing disaster experiences with attention to differential socio-economic factors. The vulnerability paradigm calls for closer attention to the socio-economic and political factors that shape inequalities, exclusion and poverty to understand who and why people are vulnerable. Since the late 1980s, gender and disaster scholars have examined how people’s socio-economic characteristics (i.e. their gender, age, ethnicity, economic and historical background, sexual orientation or religion) shape their knowledge, experiences of and behaviours in disasters and the impact disasters have on their livelihoods (Bradshaw 2013; eds. Enarson & Morrow 1998; Fothergill 1996; Schroeder 1987). Vulnerable and marginalised people are the worst affected by the occurrence of hazards because they lack access to socio-economic or political resources (i.e. savings, safe housing, decision-making power or political representation) in their everyday lives. This is overwhelmingly the case for some women, gender minorities or for ethnic groups who are discriminated against.

While this constitutes a continuous and growing research topic for Anglo-Saxon studies of vulnerability and resilience, less than a handful of academic studies published in French analyse disaster experiences with attention to gender, race or intersectionality (e.g. Cartier et al. 2017; Ezekiel 2005; Gaimard, Gateau & Ribeyre 2018; Gonon 2015; Zarowsky, Haddad & Nguyen 2013). Indeed, gender and women’s studies, just as postcolonial and critical race theories, are not often mobilised in Francophone disaster research. Francophone theoretical articles on the linkages between gender considerations and disasters are usually translated versions of more established scholarship in English literature (e.g. Enarson 2007). This might be explained by the underlying political dimension of gender and critical race studies, which, as illustrated by recent debates on the emergence of so-called ‘Islamo-leftism’ in French Universities (Le Nevé 2021), struggle to find recognition for their contribution to French academia. Attention to people’s gender or ethnicity and the resulting uncovering and/or visibility of privilege and oppression within social groups seems to come up against the logic of the *Universalisme a la française* (Gardey 2005) that aims to avoid distinctions between citizens so that they can all benefit from equal rights and adhere to universal principles. In practice, English-speaking feminist disaster scholars have long advocated for using demographic data to examine whether, how and why some people might be more affected by disasters than others. However, the fact that France forbids the collection of ethnic statistics or the usage of the concept of ‘race’, for instance, makes it particularly difficult to assess any potential differences in disaster experiences along ethnic lines occurring on French territories. The difficulty to engage freely with the gendered, raced or classed differential and intersectional dimension of people’s experience of disasters in the French context might therefore at least partially explain why many French advocates of the vulnerability paradigm in France prefer working and publishing in English and in English-speaking countries, and, by extension, be a reason for the slower emergence of the vulnerability paradigm in France than in the Anglo-Saxon context.

**FIGURE 3:**
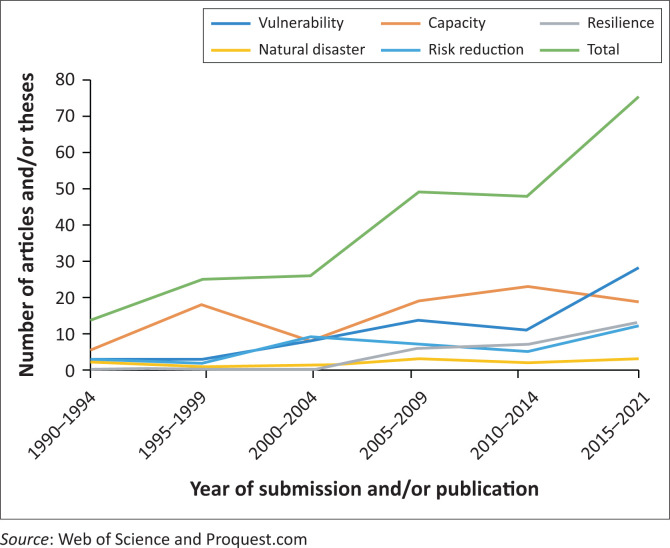
Evolution of the use of key concepts in Francophone doctoral theses and publications focusing on disaster studies in Canada (1990–2021).

This hypothesis is supported by our analysis of French-speaking disaster studies in the Canadian context. Comparing the French and the Canadian contexts is particularly useful to gain a better understanding of the role cultural and language barriers might have had on the different development of the ideas of the vulnerability paradigm in Francophone disaster studies. Canada is characterised by its bilingualism, both English and French are official languages. Its history in colonisation and its openness to immigration have resulted in a multicultural and diverse society, porous to various academic influences (Berry 2013). Francophone Canadian researchers tend to collaborate with their Anglophone colleagues and engage more with the Anglo-American disaster literature. Many are used to publishing in English to reach larger audiences. These cultural and language factors might explain why interest in disaster-related topics and the ideas of the vulnerability paradigm emerged and developed in Canada before than in France. Our bibliometric analysis thus demonstrates that interest in disasters among French-speaking researchers in Canada increased 10 years earlier than in France, with three peaks in the late 1990s (*n* = 25), between 2005 and 2009 (*n* = 49) and after 2016 (*n* = 75) (Figure 3). The term ‘natural disasters’ is seldom used in Canadian publications and theses in the field of disaster studies since 1990. However, the use of other terms reflecting the vulnerability paradigm such as ‘vulnerability’, ‘capacity’ and ‘risk reduction’ is significantly important. It seems therefore that the vulnerability paradigm might have emerged slightly before in the French-speaking part of Canada than in France.

## On the arguments and critiques put forward in 2010: A re-assessment

‘Alternatives’ called for a shift of approach in the way disasters are studied and DRR fostered in the Francophone sphere, from a strong focus on the hazard paradigm towards more emphasis on the vulnerability paradigm. While the radical and political criticisms formulated and institutionalised in the 1970s in the Anglo-Saxon World penetrated the Latin American and South-Asian contexts as early as the mid-1990s (see Cabane & Revet 2015), this paradigm shift took much longer in the French context. A few articles published by French authors since the 1970s have developed strong arguments on the socio-structural root causes of disasters (e.g., writings of neo-Marxist economic anthropologist and Africanist Claude Meillassoux on famine in Africa) (Meillassoux 1974). However, these writings seem to have not influenced disaster studies in the Francophone literature as much as they did in the Anglophone spheres. In the previous section we suggest that language barrier and the French republican and assimilationist philosophical tradition, rejecting the consideration of individual specificities, might have slowed down this process and explain why some of the critiques and arguments at the heart of the vulnerability paradigm have still not fully made their way in the French disaster literature. Furthermore, the term ‘natural disaster’, as a marker of the hazard paradigm, continues to be used in French publications and theses. All these suggest that the critiques and arguments of ‘Alternatives’ are still relevant today.

At the same time, it appears that some of the ideas advanced in the ‘Alternatives’ have continued to take root in the Francophone disaster literature. Both emerging and more established Francophone researchers have increasingly emphasised that disasters result from the unfair distribution of resources and power in society. Nowadays, a large majority of Francophone researchers are applying some version of the vulnerability paradigm. In pushing for this paradigm shift, researchers have sometimes claimed to be critical and radical. However, one may wonder whether these contemporary users of the vulnerability paradigm have actually taken on the challenges set up almost 50 years ago (Gaillard 2021), especially with regard to its aim of challenging Western neo-colonial and technocratic approaches to disasters and DRR. Currently, Francophone disaster scholars, such as their Anglo-Saxon colleagues, continue to be dominated by some of the same Western epistemologies that the vulnerability paradigm wanted to challenge in the first place (Gaillard 2019).

With regard to research conducted in France, while particularly exposed to numerous hazards, French overseas territories (La Réunion, French Antilles, New Caledonia, French Polynesia, etc.) remain understudied in comparison to metropolitan France. Although this tendency is slowly reversing (Moatty, Grancher & Duvat 2021), it necessarily resonates with France’s historical and institutional disdain for its former colonies. In the French collective imagination, the ‘French Republic’ remains today usually constituted of the European mainland and Corsica. This is what Ferdinand (2018:119) calls the ‘single geographical imaginary of France’. This simplistic geographical representation of the French territory tends to serve a broader purpose – that of sustaining the portrayal of inhabitants of the French Overseas Territories as a population ‘in development’, in opposition to the French mainland, seen as an independent European entity that ‘extends its benevolent hand to care for its overseas citizens, for which the latter should be grateful’ (Ferdinand 2018:130). In addition to sustaining a Western neo-colonial and technocratic vision of the world, this narrative tends to limit the responsibility of the French government for the persistent inequalities between the overseas and mainland France. For instance, DRR in Reunion and Mayotte Islands was characterised, at least until 2010, by a top-down management of mainland policies based on the assumption of similar vulnerabilities despite obvious differences between the two socio-economic contexts (Le Masson & Kelman 2011).

**FIGURE 4:**
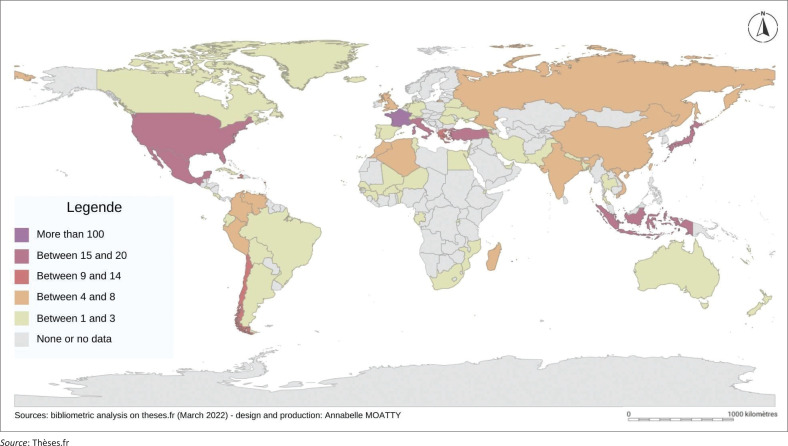
Geographical distribution of French doctoral research’s case studies between 1985 and 2021.

Our bibliographic analysis also demonstrates that an important number of French disaster scholars and young researchers focus their research on case studies outside of France. Figure 4 shows that with the exception of France and Italy, the list of countries having stirred the greatest interest of French PhD candidates between 1985 and 2021 (Indonesia, USA, Japan, Mexico and Haiti) largely overlaps with the classical list of ‘major’ disasters having attracted great attention of scholars worldwide over the past 20 years: Boxing Day Earthquake and tsunami (Indonesia, 2004), Hurricane Katrina (USA, 2005), Jakarta floods (Indonesia, 2007), Mount Merapi eruptions (Indonesia, 2010), Haitian earthquake (Haïti, 2010) and Tohoku Earthquake and Tsunami (Japan, 2011). In this sense, French scholars tend to contribute to the globally unbalanced distribution of authorship in the disaster field with Western scholars dominating the field whatever the location of the hazard (Gaillard 2019). This focus on large-scale disasters is largely linked to research funding modalities that tend to favour major disasters over the study of more frequent events of lesser magnitude. This might also contribute to sustaining the problematic dynamic of the ‘gold rush’ (Gaillard & Gomez 2015) that is:

[*T*]he ‘imperative’ of collecting ‘perishable’ data often resulting in an influx of Western researchers, frequently with limited knowledge of the disaster-affected areas and with insufficient time to collect enough background information, to learn the local language, and to get to know the local culture, leading regularly to misconceptions. (Gaillard 2019:5)

This is particularly problematic because many key concepts of disaster studies, like vulnerability or resilience, reflect Eurocentric and Western approaches to studying disasters and do not necessarily translate or apply to specific contexts where different epistemologies and ontologies might apply.

‘Vulnerability’ and cognate concepts have been rolled out across continents, including in places where they cannot be translated into local languages and in contexts where cultures, history, worldviews, knowledge and local problematics differ (Le Dé & Gaillard 2022). Such supposedly universal concepts tend to be imposed upon people who struggle to make sense of their meanings. This approach is problematic with regard to the diverse realities of the world and people’s numerous perspectives on events that may look hazardous or harmful through a Western lens (Hewitt 1983). The culture and visions of both researchers and practitioners using Eurocentric paradigms hold implicit and explicit assumptions about what is essential in the everyday life in a locality, what must be protected and according to whom and for whom. Similarly, most of the methodologies that have come with the increasing popularity of disaster studies reflect Eurocentric and Western epistemologies. As a result, the Eurocentric and Western worldview of disaster risk management is dominant in international DRR guidelines in comparison with other frameworks or approaches such as indigenous risk management worldviews.

Different authors describe how dominant Western-informed DRR policy and practice ignore the cultural contexts in which hazards and risks occur and highlight the negative consequences of such ignorance for successful and sustainable DRR (Ali et al. 2021; Dake 1992). Despite the fact that there is a large literature about the importance and advantages of incorporating Indigenous Ecological Knowledge (IEK) systems into DRR (Chen & Cheng 2020; Cochran et al. 2013), traditional and indigenous views that reflect locally grounded ontologies and epistemologies remain poorly acknowledged in the practice of reducing risk. Understanding disasters and reducing disaster risk demands a better integration and interpretation of the views and experiences of local people. This entails a shift in the way we apprehend, assess and interpret disasters and people’s experiences, with locals playing a central role in the knowledge production, including on the use of local concepts and solutions to address disaster risk.

## Alternatives for sustained disaster risk reduction: Where to?

In 2010, ‘Alternatives’ voiced the need for a shift in the way disasters were researched and DRR fostered in the Francophone world where the hazard paradigm was still prominent. The article called for an approach that reframes disasters and DRR in the context of people’s everyday life, with the need to better understand vulnerabilities, marginalisation processes and focusing on local people’s knowledge, resources and skills. Twelve years later, these ideas have, to some extent, progressed in the Francophone literature. The vulnerability paradigm has gained traction, and there are now more Francophone researchers coming from social sciences and humanities than physical sciences and engineering. Nonetheless, there is a need for a constant re-assessment of our practices, approaches and how we study disasters.

As of today, Francophone disaster studies, such as their Anglo-Saxon counterparts, remain largely dominated by some of the same Western epistemologies that the vulnerability paradigm hoped to challenge in the first place (Gaillard 2019). Supporters of the vulnerability paradigm critique the disproportionate interest of disaster scholars for rare, large scale and extreme events, at the expense of small scale, everyday disasters that tend to be of greater interest to local people (Shrestha & Gaillard 2013). In the Francophone context, just as in the rest of the world, disaster scholars and young doctorates keep devoting particular attention to large-scale ‘sensational’ hazards. Moving forward, there is a need for more research documenting smaller scale and less visible disasters as well as the processes that lead to increasing vulnerability. Local Francophone researchers, who are more experienced and knowledgeable about the local contexts, should thus play a central role in studying these disasters and risk accumulation processes. For instance, PhD candidates and more established researchers from French overseas territories such as French Antilles, New Caledonia, La Réunion, French Polynesia should play a greater role in researching their own localities and be supported in the role of research project leaders, with the support of their colleagues from mainland France. However, the academic publication and research funding system are currently not set up to support more pluralistic views and local leadership in the study of disasters. Emerging academics, in particular, must publish in high-impact factor journals as fast as possible following large-scale events. Publishing first is likely to generate attention from the media and contribute to the reputation of scholars’ institutions, while, frequently, also encouraging practices that are ethically dubious (Gomez & Hart 2003).

The study of disasters requires better integration and interpretation of the viewpoints, experiences and worldviews from local people. Scholars and practitioners have long encouraged the participation of local people on matters that affect their lives (Chambers 2003; Maskrey 1984, 1989). They have contributed to the rise of participatory pluralism as a plausible paradigm alternative to Eurocentric and Western approaches to DRR (Chambers 2007). Central to people’s participation is the idea that power and knowledge are strongly connected (Freire 1970; Said 1978). This alternative paradigm acknowledges that while they might be vulnerable, people can also be valuable researchers. Creating a body of knowledge is a precondition to make use of power, and knowledge reflects power relationships (Foucault 1975). Participatory approaches and methods therefore aim to empower local people within the decision-making process so they can shape or control the decisions intended to foster DRR (Saxena 1998). Participatory pluralism recognises that people and local communities have capacities – in other words, skills, knowledge and resources – and that building upon such capacities is a precondition for effective and sustainable DRR. Unfortunately, participatory approaches and methods are seldom used in the Francophone disaster literature (approximately 5% of publications found in the Web of Science). Furthermore, initiatives based on participatory approaches are often ‘quick and dirty’ [in Chambers’ words] and facilitated by DRR agencies whose agenda may differ from that of local people (Cooke & Kothari 2001). More appropriate participatory methods are required such as long-term participant observation by native speakers or at least researchers fluent in local vernaculars.

Studying disasters and looking at ways to foster DRR require questioning power and power relations. What disasters are studied? How, by whom and for whom are they approached? Disaster research requires more diverse ontologies and epistemologies to be grounded and pertinent locally. Local Francophone researchers in different parts of the world should be encouraged to utilise concepts, epistemologies, ontologies and methodologies that suit their local context and reflect local realities (Le Dé & Gaillard 2022). There are plenty of brilliant Francophone researchers in Africa, Latin America, Southeast Asia and the Pacific to lead this process and raise consciousness among their colleagues. Consciousness is essential to resist the domination of Western scholarship and draw upon local and traditional knowledge, skills and resources (Freire 1970). Turning over power relations in researching disasters necessitates reclaiming the political dimension of disasters. Top-down and technocratic solutions for DRR continue to dominate because disasters are mainly seen through the reductionist and positivist Eurocentric and Western lens. Questioning whose knowledge and studies benefit whom should be at the core of a more politically grounded disaster research agenda. Sharing power with local researchers so that they lead disaster projects and studies is one key step in this direction (Wisner & Lavell 2017).

## Footnotes

1 Web of Science’s database goes back to 1955. However, the number of publications was very low until the 1980s: 6 publications/year on average for the period 1955–1985 while 67 publications/year were published on average for the period 1985–2021.
